# Massachusetts Veterinary Association

**Published:** 1897-06

**Authors:** Henry S. Lewis

**Affiliations:** Secretary


Secretary.

### MASSACHUSETTS VETERINARY ASSOCIATION.

THE regular monthly meeting was held at No. 19 Boylston Place, Boston, on Wednesday evening, March 24, 1897. Meeting was called to order by Dr. Winslow Members present: Drs. Burr, Connor, Dyer, Lee, Lewis, McLaughlin, Winchester, Winslow, and Stickney. John M. Armstrong, M.D., was elected a member of the Association.

The Chair appointed Drs. Peters, Blackwood, and McLaughlin a committee to make arrangements for our thirteenth annual meeting and banquet on April 28th.

HENRY S. LEWIS,
Secretary.

